# The role and mechanisms of gut microbiota in diabetic nephropathy, diabetic retinopathy and cardiovascular diseases

**DOI:** 10.3389/fmicb.2022.977187

**Published:** 2022-08-18

**Authors:** Qiulan Lv, Zhiyuan Li, Aihua Sui, Xiaomin Yang, Yafei Han, Ruyong Yao

**Affiliations:** Medical Research Center, The Affiliated Hospital of Qingdao University, Qingdao, China

**Keywords:** T2DM-related complications, gut microbiota, microbial metabolites, intestinal barrier, immunity, microbiological therapy

## Abstract

Type 2 diabetes mellitus (T2DM) and T2DM-related complications [such as retinopathy, nephropathy, and cardiovascular diseases (CVDs)] are the most prevalent metabolic diseases. Intriguingly, overwhelming findings have shown a strong association of the gut microbiome with the etiology of these diseases, including the role of aberrant gut bacterial metabolites, increased intestinal permeability, and pathogenic immune function affecting host metabolism. Thus, deciphering the specific microbiota, metabolites, and the related mechanisms to T2DM-related complications by combined analyses of metagenomics and metabolomics data can lead to an innovative strategy for the treatment of these diseases. Accordingly, this review highlights the advanced knowledge about the characteristics of the gut microbiota in T2DM-related complications and how it can be associated with the pathogenesis of these diseases. Also, recent studies providing a new perspective on microbiota-targeted therapies are included.

## Introduction

Type 2 diabetes mellitus (T2DM), a growing epidemic disease, represents ∼90% of all diabetes mellitus (DM) cases and is one of the most prevalent metabolic diseases affecting > 400 million people worldwide (Carracher et al., 2018). More importantly, the secondary complications burden patients with poor quality of life, high costs, and constrained employment opportunities. In general, genetic susceptibility and lifestyle factors are the major culprits of T2DM (Scott et al., 2013). Although the development of T2DM is intricate and heterogeneous, low-grade inflammation is suggested to accompany the progress of T2DM (Després et al., 2008; Donath and Shoelson, 2011) causing the development of secondary complications, including retinopathy, nephropathy, neuropathy, and cardiovascular diseases (CVDs) (Cusi et al., 2017; Beli et al., 2018; Kikuchi et al., 2019; Attaye et al., 2020). Recent studies showed that T2DM complications are mutually dependent sharing common pathways (Brownrigg et al., 2016; Yousri et al., 2022). Thus, identifying the root cause of these common pathways that induce low-grade inflammation in T2DM can be an effective approach for the clinical treatment of T2DM and related diseases.

The host gut microbiota, with > 150-fold genetic material than the host, functions as a full-fledge endocrine organ affecting the host nervous system, metabolic diseases, microvascular diseases, ulcerative colitis, and nephropathy (Qi et al., 2021). Furthermore, the rapid development of high-throughput sequencing technology and the implementation of the human microbiome sequencing project have enabled increasingly accessible and efficient microbiome studies in T2DM highlighting the importance of the gut microbiota-organs axis as a promising target. Recently, population-based studies showed that the composition and function of the gut microbiota are altered in subjects with prediabetes or T2DM (Karlsson et al., 2013a). Rodrigues et al. (2021) applied a data-driven approach to model host-microbiome interactions and showed that bacterial species Lactobacillus johnsonii, Lactobacillus gasseri, Romboutsiailealis, and Ruminococcusgnavus contribute to T2DM-related systemic changes. Furthermore, arrhythmic bacterial signatures were found to be useful for the classification and prediction of T2DM (Reitmeier et al., 2020). T2DM showed characteristics of microbiota dysbiosis and compromised intestinal barrier, which increases the systemic circulation of lipopolysaccharide (LPS) triggering systemic inflammation (Qin et al., 2012; Karlsson et al., 2013b). Microbiota dysbiosis not only mediates a maladaptive immune response in the intestine but also facilitates crosstalk between the intestinal and host organ immune cells during T2DM contributing to low-grade inflammation (Karlsson et al., 2013b; Zhernakova et al., 2016). Thus, deciphering microbiota signatures specific to T2DM complications can be a key to therapeutic strategy in future.

The mechanisms through which gut microbiota regulates host diseases involve migration to distant sites, changing microbial metabolites, promoting intestinal barrier dysfunction, and controlling the immune system function. The role and mechanisms of gut microbiota in obesity and T2DM have been widely accepted (Canfora et al., 2019; Aron-Wisnewsky et al., 2020). In this article, we mainly discuss the recent advances in gut microbiota signatures and mechanistic insights into the progress of diabetic kidney, retinopathy, and CVDs. Then, we focus on how the gut microbiota regulates the progress of these diseases from the viewpoint of microbial metabolites, intestinal barrier, and microbiota-immune crosstalk. Finally, we discuss the recent research that provides new insights into microbial therapeutics.

## Bacterial dysbiosis in diabetic complications

Microbiota mediates crosstalk between the intestine and host organs by migrating to distant sites affecting the progression of diseases. A metagenome-wide association study showed that the patients with T2DM exhibited a decrease in the abundance of butyrate-producing bacteria and an increase in opportunistic pathogens (Qin et al., 2012). Bacterial dysbiosis is one of the key features of T2DM and has been reviewed elsewhere (Fan and Pedersen, 2021). Here, we emphatically review the alterations of gut microbiota that affect the development of diabetes-associated complications.

### Dysbacteriosis in diabetic nephropathy

Diabetic nephropathy (DN), characterized by glomerular damage, is frequently regarded as the leading cause of end-stage renal disease. Studies showed that 70% of DM patients have abnormal renal structure (Vestra et al., 2000). Also, insulin resistance, hyperglycemia, and chronic inflammation are the principal factors aggravating the progress of DN. Growing evidence suggests that aberrant gut microbiota may be a crucial factor in the pathophysiology of DN. The host gut microbiota changes from the higher relative abundance of Firmicutes and lower abundance of Bacteroidotato lower Firmicutes, higher Bacteroidota and Proteobacteria as well as high ratio of Bacteroidota -to-Firmicutes predominantly showing an increase in glucose level and insulin resistance (Larsen et al., 2010). Phylum Firmicutes and Bacteroidota are the predominantly autochthonous microbiome in the intestine, while Proteobacteria, Actinobacteriota, Fusobacteriota and Verrucomicrobiota represent fewer taxa (Eckburg et al., 2005; Niches, 2011). The autochthonous gut microbiome evolved with specialized defense against endogenous antimicrobial peptides. Thus, a disturbing proportion of Firmicutes and Bacteroidota has been recognized as a potential diagnostic signature of intestinal dysbiosis that contributes to the progress of the disease (Eckburg et al., 2005). Phylum ratios Bacteroidota-to-Actinobacteriota and Bacteroidota-to-Firmicutes were found significantly increased in DN rats (Zhao et al., 2020). Furthermore, the microbiota that increased in chronic kidney disease were also increased in DN rats (f-Enterobacteriaceae and g-Ruminococcus) (Kanbay et al., 2018; Zhao et al., 2020). Similarly, another study in kidney-impaired db/db mice showed a significant reduced in Phylum Proteobacteria, Verrucomicrobiota and Epsilonbacteraeota. The genus Bacteroides, Eubacterium, and Roseburia was increased, while the abundance of *Desulfovibrio* was reduced (Li et al., 2020a).

Albuminuria, a hallmark of early DN, is one of the dominant clinical manifestations during the development of this disease. A study showed that families Lactobacillaceae, Enterobacteriaceae, unclassified Clostridiales, and Streptococcaceae were increased and strongly correlated with elevated albuminuria in diabetes (Li et al., 2020b). Similarly, genus Abiotrophia and family Peptococcaceae showed a positive correlation with albuminuria levels and the progression of DN (Zhang et al., 2021). DN has also been closely linked with the activation of proinflammatory pathways. Gut microbiota-derived short-chain fatty acids (SCFAs) play important roles in regulating the immune response and inflammation. The decrease in the abundance of SCFAs producing bacteria changed the gut environment to the pro-inflammatory environment in tubulointerstitial injury. SCFA producing bacteria such as species Bacteroides acidifaciens, genus Ruminococcus and Rikenella were found reduced in diabetic rats with tubulointerstitial injury, while acetate-producing genus Lactobacillus and Phascolarctobacterium were significantly elevated (Hu et al., 2020; Li et al., 2020b). Evidence showed that dietary fiber can protect against DN through enrichment of SCFA-producing bacteria, while serum glucose levels still remained higher (Li et al., 2020b). Depletion of gut microbiota or transplantation of control fecal microbiota in DN was shown to alleviate tubulointerstitial injury by improving dysregulation of cholesterol homeostasis (Hu et al., 2020). In all, these studies indicate that gut microbiota plays a vital role in DN. The possible mechanisms may be the translocation of pathological microbiota to metabolic sites through the compromised intestinal barrier that might directly perturb the intestinal and local tissue immune landscapes thereby promoting the progress of DN. However, rare studies attempted to discriminate the discrepancy between DN and T2DM without nephropathy. Tao et al. (2019) showed that genus Escherichia-Shigella and Prevotella-9 can be used to distinguish T2DM patients with or without renal complications. Identifying the specific microbiota in DN could provide a better framework to understand the progress of DN. However, there is a lack of such studies. Further research is required to pay more attention to the changes in microbiota in T2DM without nephropathy and that at early stages of DN. Notably, different studies reported contradictory levels of some microbiota. These can be ascribed to considerable sample disparity in terms of bacterial cell density of stools and inter-individual variations (Vandeputte et al., 2017).

### Dysbacteriosis in diabetic retinopathy

Diabetic retinopathy (DR), also called microvascular disease, is a major complication of diabetes causing blindness and vision impairment. About 35% of T2DM patients are affected by DR (Dao et al., 2021). Mechanisms underlying the pathogenesis of DR are multifactorial and intricate. Dyslipidemia, chronic inflammation, and high blood pressure are recognized as the major risk factors for developing DR (Mitchell et al., 2018). Emerging research suggests that changes in gut microbiota may play a key role in the progression of host disease. Notoriously, Gut microbiome and the products can transport to the liver through portal vein, while the bile and antibodies from the liver remodeling gut microbiome. The bidirectional relationship between gut and the liver affect a range of hepatic diseases, known as “gut-liver axis” (Albillos et al., 2020). The gut and the kidney also share a bidirectional relationship of synergy, namely the “gut-kidney axis.” Microbiota-derived metabolites contributes to uremic toxins. Kidney epithelial cells can sense gut-derived metabolites and maintain body homeostasis (Jansen et al., 2019). Similarly, Brain and gut shares a reciprocal communication. Gut microbiome can influence enteric nervous system via producing local neurotransmitters and bioactive form of catecholamines. In reverse, the enteric nervous system can bifacially communicate with central nervous system and then regulate gut microbiome, thereby forming the “gut–brain axis” (Van De Wouw et al., 2017; Zhao et al., 2021). Recently studies showed that gut microbiota dysbiosis can contribute to eye diseases (Rowan and Taylor, 2018; Janowitz et al., 2019) proposing the “gut-retina axis.” Diabetic patients with delayed gastric emptying had a higher risk of retinopathy, while bariatric surgery reduced the incidence of DR (Parkman et al., 2019; Singh et al., 2021). Bariatric surgery can change microbiota structure and enhance intestinal barrier function facilitating weight loss and improving glycemic control, which is a key factor in DR (Koutoukidis et al., 2022; Scheithauer et al., 2022). Recently, a direct relationship between gut microbiota and DR was shown. Gut microbial dysbiosis was found in DR patients; the genus Bifidobacterium and Lactobacillus were elevated, while Escherichia-Shigella, Faecalibacterium, Eubacterium_hallii_group and Clostridium were decreased. Interestingly, compared with DM patients, families such as Oscillospiraceae, Christensenellaceae, Acidaminococcaceae, and Anaerovoracaceae were significantly enriched in DR patients. Furthermore, twenty-two genus mostly belonging to Acidaminococcaceae, Muribaculacea, Atopobiaceae and noranko-Coriobacteriales families were only detected in the DR group, indicating their specific role in DR (Huang et al., 2021). A similar study was also reported by Das et al. (2021) the major phyla Bacteroidota and Actinobacteriota were significantly lower in DR patients compared to healthy individuals. The pro-and anti-inflammatory gut microbiome was disturbed. The anti-inflammatory genera Roseburia, Lachnospira, Blautia, Faecalibacterium, Clostridium, Butyrivibrio, and Bifidobacterium were decreased, while pathogen bacteria Escherichia, Enterobacter, Cloacibacillus, and Enterococcus were increased in DR. Compared to DM, thirteen genera (Bifidobacterium, Mitsuokella, Streptococcus, Klebsiella, Desulfovibrio, Lachnobacterium, Erwinia, Treponema, Methanobrevibacter, Haemophilus, Asteroleplasma, Anaerovibrioand Weissella) were decreased, and five genera (Phascolarctobacterium, Alistipes, Shigella, Cloacibacillus, and Enterococcus) were increased, which may be the crucial microbiota for developing DR. Notably, genus Cloacibacillus and Enterococcus were only detected in DR. However, another study showed that the genera Faecalibacterium, Roseburia, Lachnospira, and Romboutsia were increased and species Akkermansia muciniphila was decreased in DR. Compared to DM patients, the abundance of genera Prevotella, Faecalibacterium, Subdoligranulum, Agathobacteria, and Olsenella were increased, while genus Bacillus, Veillonella and Pantoea were reduced in DR (Zhou et al., 2021).

The role of gut microbiota in DR was further highlighted by Beli et al. (2018) showed that intermittent fasting can prevent retinopathy by restructuring the gut microbiome by increasing Firmicutes and decreasing Bacteroidota and Verrucomicrobiota in db/db Mice. Interestingly, Dao et al. (2021) showed that high-fat diets (HFD) can directly modulate the retinal transcriptome independent of the gut microbiome. There is significant variation in the bacterial genera described in different studies that may be ascribed to inter-individual variations, differences in employed methodologies, and staging of DR. However, all studies manifest that dysbacteriosis is the common feature of DR.

### Dysbacteriosis in diabetic cardiovascular diseases

Cardiovascular diseases are a cluster of disorders of the heart and blood vessels with clinically manifest such as atherosclerosis, vascular inflammation, myocardial ischemia, myocardial infarction, and heart failure (Kosiborod et al., 2018). Diabetes increases the risk of CVD by 2–3-fold; ∼65% of diabetic patients die from CVD complications (Rao Kondapally Seshasai et al., 2011). The bacterial taxa observed in atherosclerotic plaques were also present in the gut, indicating the vital role of gut microbiota in the development of CVD (Koren et al., 2011). Previous studies showed the occurrence of gut microbiota dysbiosis in CVD (Nagatomo and Tang, 2015; Jie et al., 2017; Pm et al., 2021). The gut anti-inflammatory SCFA producing bacteria profile changed into the pathogen microbiota that promotes inflammation disturbing the immunological balance. Meanwhile, the decreased abundance of Akkermansia muciniphila also contributed to the compromised intestinal barrier. Bacteria dysbiosis in CVD is mainly manifested as a decrease of phyum Bacteroidota and increase of Firmicutes. Furthermore, the SCFA butyrate microbiota such as Faecalibacterium, Eubacteriumrectale, Roseburia, and Eubacterium were reduced in CVD, while pathogen microbiota including Streptococcus, Escherichia-Shigella and Enterococcus increase (Karlsson et al., 2012; Jie et al., 2017; Qi et al., 2018). A concept of the “heart-gut-microbiota axis” has been proposed as a new target for atherosclerosis therapy. However, the direct evidence for the gut microbiota signature in diabetic CVDs is largely unclear. A cross-sectional study showed high levels of Firmicutes and low levels of Bacteroidota and Bacteroides was positive correlation with an increased risk of left ventricular hypertrophy and diastolic dysfunction in diabetes (Tsai, 2021). Similarly, Lee et al. (2018) showed a decrease of species Akkermansia muciniphila and an increase of genus Enterococcus in diabetic mice with vascular dysfunction. Also, the levels of Proteobacteria and Firmicutes, and Firmicutes-to-Bacteroidota ratio were found to be significantly correlated with vascular outcomes. Furthermore, a study showed that mechanistically dapagliflozin improves vascular dysfunction by subtly altering microbiota composition (Lee et al., 2018). Pathogen infection is a strong risk factor for CVD (Cowan et al., 2018). In diabetes, hyperglycemia-mediated barrier disruption enhances the dissemination of enteric infection and increases susceptibility to pathogenic microbes, which may be another key factor for the progression of CVD (Thaiss et al., 2018). Consistently, pathogenic microbes (Staphylococcus, Klebsiella, Mycobacterium) and gram-positive bacteria (specifically the species Clostridium coccoides and Atopobium cluster) were found elevated in the blood of T2DM patients (Smit et al., 2016). Furthermore, higher levels of *Staphylococcus* spp. were also detected in blood cultures from CVD patients (Dinakaran et al., 2012). It could be that gut microbiota translocates into the host blood and colonizes in atherosclerotic plaques and cardiac tissues causing systemic inflammation and subsequently CVD.

## Bacterial metabolites affect diabetic complications

**FIGURE 1:**
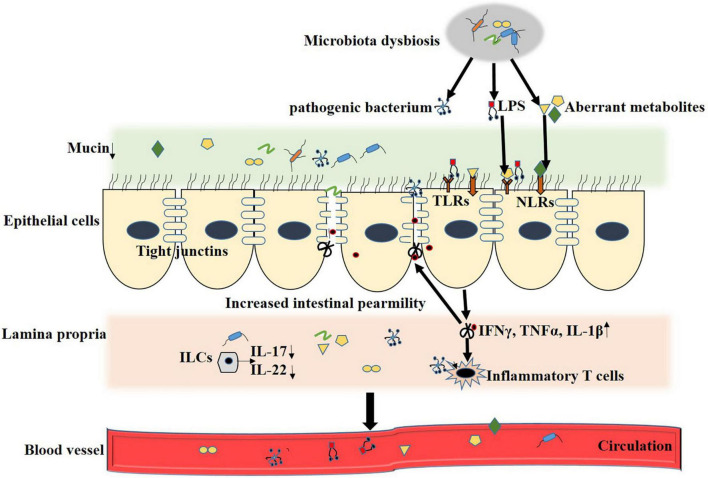
Microbial metabolites regulate T2DM complication. Loss of integrity makes the gut epithelium more vulnerable to allow pathogenic bacterium and metabolites translocate into systermic circulating. Disturbed gut microbiota result in decreased systemic short-chain fatty acids (SCFA) concentrations, which, in turn, promotes inflamation and decreases glucagon-like peptide 1 (GLP-1) secretion by acting on G protein-coupled receptor (GPCRs). Elevated trimethylamine (TMA) in gut translocate to liver through the leaked intestinal barrier and is oxidized into trimethylamine N-oxide (TMAO) by Flavin containing monooxygenase3 (FMO3), which further enter into systemic circulation and contributes to insulin resistance, diabetic nephropathy and CVD. Secondary bile acids are involved in multiple hormonal regulatory by antagonizing farnesoid X receptor (FXR) and G-protein coupled bile acid receptor (TGR5) and contributed to development of diabetic retinopath and CVD. Elevated circulation of lipopolysaccharide (LPS) trigger innate immunity and construct a chronic inflammation status by activating Toll like receptors (TLRs) and NOD-like receptors (NLRs), which is the culprit of T2DM related complication.

In general, DM with microbial dysbiosis accompanies the disturbance in bacterial metabolites, which mediate crosstalk between the intestine and remote organs. Several reports demonstrated that microbiota regulated metabolic disease progression immensely depends on the change in microbiota metabolites (Koh et al., 2018; Krisko et al., 2020). Gut microbial metabolites directly interact with intestinal epithelial cells and immune cells impacting the host’s health. An array of plasma metabolites were altered in T2DM, even in the early stages. In the following sections, we selectively review some of the reported intestinal microbial metabolites that affect DN, DR, and CVDs. The mechanism of microbial metabolites affecting diabetic complications is shown in Figure 1.

### Bacterial metabolites affect diabetic nephropathy

SCFAs, including butyrate, propionate, and acetate, have been recognized as key factors for regulating insulin signaling, lipogenesis, and inflammation. These also play vital roles in the progress of metabolic diseases. SCFAs could alleviate DN by regulating the regulatory T cells (Tregs). Studies showed that T2DM patients with nephropathy had lower levels of Tregs than those without renal injury. Also, depleting Tregs levels worsen insulin resistance and albuminuria. SCFAs can alleviate systemic inflammation by activating Tregs (Corthay, 2009; Eller et al., 2011). A study showed that exogenous administration of SCFAs can promote Treg production (Yao et al., 2022), which could be a regular phenomenon. However, whether SFAC promoting Treg production relate to the changes in the microbiome is unknown. Mo et al. (2021) showed that fecal microbiota transplantation (FMT) from high SCFAs mice could alleviate DSS-induced colitis by enhancing the levels of Tregs and serum anti-inflammatory cytokines, which is related to the reconstruction of microbial communities, suggesting that gut microbiota can modulate Treg production. Furthermore, compelling evidence indicated that dietary fiber can improve kidney disease by regulating the production of SCFAs. Dietary fiber protects against DN by promoting SCFA-producing bacteria and systemic SCFAs concentration through the GPR43 and GPR109A metabolite receptors. Notably, SCFA supplementation could protect against DN (Li et al., 2020a,b). Similarly, butyrate levels were found reduced in DN patients, and supplementation with butyrate suppressed autophagy and oxidative stress by activating the PI3K/Akt/mTOR pathway in DN mice (Tang et al., 2022). However, other studies demonstrated conflicting results. Li et al. (2020b) showed that gut microbiota-derived acetate contributed to the development of DN by activating the G protein-coupled receptor 43 (GPR43). The mechanism may be that superfluous acetate activates the GPR43 pathway inhibiting AMPKα activity, which induces the dysregulation of cholesterol homeostasis and insulin signaling, ultimately facilitating the progression of DN (Li et al., 2020b). Antibiotics or FMT treatment could alleviate kidney injury that was accompanied by a decrease in serum acetate levels (Hu et al., 2020; Lu et al., 2020). Unlike butyrate and propionate, acetate may predominantly have obesogenic properties since it acts as a substrate for hepatic and adipocyte lipogenesis. Increased acetate production induced by an altered gut microbiota promotes hyperphagia and fat storage by increasing glucose-stimulated insulin secretion (Perry et al., 2016; Xue et al., 2016).

Indoxyl sulfate (IS), phenyl sulfate (PS), and trimethylamine N-oxide (TMAO) are recognized as the prototypical microbiota-derived uremic toxins that contribute to chronic kidney disease. Interestingly, TMAO, IS, and PS were not detectable in germ-free or antibiotic-treated mice, indicating an obligatory role of gut microbiota (Koeth et al., 2015). IS, a newly identified aryl hydrocarbon receptor ligand produced by gut microbiota, has been found to accumulate in the serum of chronic kidney disease patients and contributes to renal inflammation and fibrosis (Brito et al., 2017; Hung et al., 2017). Diabetic patients with macroalbuminuria exhibited higher plasma concentrations of IS and L-citrulline while lower levels of tryptophan (Winther et al., 2020). Likewise, increased levels of IS were also found in DN rats, and reducing IS levels inhibited diabetic renal injury (Zhao et al., 2020). In a diabetic patient cohort, PS levels were significantly correlated with albumin-to-creatinine ratio and predicted its 2-year progression to deterioration in chronic kidney disease patients, especially with microalbuminuria. Similarly, in experimental models of diabetic rats, PS levels increased with the progress of DN, while decreased in rats with limited proteinuria. PS supplementation rendered cells vulnerable to oxidative stress and induced albuminuria. Furthermore, inhibition of tyrosine phenol-lyase, a bacterial enzyme responsible for the synthesis of PS, reduced albuminuria in diabetic mice indicating that PS can be a therapeutic target in DN. The microbial genera Adlercreutzia and Erysipelotrichaceae unclassified exhibited a significant correlation with PS levels and were increased in DN (Kikuchi et al., 2019). Mechanistically, PS and IS may contribute to DN by provoking oxidative stress, and pro-inflammatory and pro-fibrotic responses in tubular cells, thereby aggravating the progress of DN (Bolati et al., 2013). Overwhelming clinical studies revealed increased TMAO levels in diabetic patients, which also strongly correlated to renal dysfunction (Stubbs et al., 2016; Winther et al., 2019). A recent study showed significantly increased TMAO levels in DN rats; furthermore, TMAO supplementation accelerated the development of chronic kidney disease by activating the nucleotide binding oligomerization domain like receptor family Pyrin domain protein 3 (NLRP3) inflammasome, and provoking the release of interleukin (IL)-1β and IL-18 (Fang et al., 2021).

Bile acids (BAs) are synthesized in the liver and further metabolized by gut microbiota to generate secondary BAs (Dawson and Karpen, 2015). Secondary BAs can be involved in DN by antagonizing the nuclear membrane farnesoid X receptor (FXR) and the G protein-coupled receptor 5 (TGR5) in the intestine. A study showed that secondary BAs were involved in the progress of chronic kidney disease (Wang et al., 2020). In DN rats, the levels of total bile acids (TBA), cholic acids (CAs), and deoxycholic acids (DCAs) were greatly increased. Kidney injury was alleviated by altering the CA-to-TCA (taurocholic acid) and DCA-to-CA ratios (Zhao et al., 2018). TBA, FXR agonistic ligand taurocholic acid (TCA), and β-muricholic acid (β-MCA) were dramatically increased in DN mice than in control mice (Wei et al., 2021). Increased TCA and β-MCA levels can inhibit FXR activation. Evidence showed that FXR activation is indispensable for preventing the progression of DN. Global FXR knockout exacerbated renal injury in type 1 diabetes (Wang et al., 2010). Thus, elevated TCA and β-MCA levels can increase the risk of DN by inhibiting FXR activation. Nidufexor, an FXR agonistic, has been advanced to phase 2 human clinical trials for DN treatment (Chianelli et al., 2020). The ATP-binding cassette transporters G8 plays a vital role in bile acid secretion and was shown to be associated with the incidence of kidney disease in T2DM patients (Miao et al., 2015). Wang et al. (2016) showed that TGR5 was upregulated in kidney specimens from DN patients and could be associated with DN progression. TGR5 agonist INT-777 alleviated DN by preventing renal oxidative stress and lipid accumulation in diabetic db/db mice. Similarly, another study showed that TGR5 activation ameliorated DN potentially by inhibiting the sphingosine 1-phosphate/sphingosine 1-phosphate receptor 2 signaling, reducing intercellular adhesion molecule-1, and then the expressions of transforming growth factorbeta1 and fibronectin (Yang et al., 2016).

Other microbiota-related metabolites such as hippuric acid, L-(-)-3-phenylacetic acid, trans-3-hydroxy-cinnamate, dihydro-3-coumaric acid, and indole-3 acetic acid also showed a positive correlation with the progression of diabetic kidney disease, whereas L-tryptophan exhibited a negative correlation (Zhang et al., 2021).

### Bacterial metabolites affect diabetic retinopathy

A multiplatform-based metabolomics study comprehensively revealed that the metabolites dysregulation was associated with the onset and progression of DR. 12-hydroxyeicosatetraenoic acid and 2-piperidone were associated with the progression of DR (Xuan et al., 2020). Notably, the synthesis of 12-hydroxyeicosatetraenoic acid is modulated by gut microbiota, indicating the crucial role of microbiota in DR (Abot et al., 2021). Microbial metabolites in DR significantly differed from those in healthy individuals and DM patients. Levels of carnosine, succinate, nicotinic acid, and niacinamide were found significantly lower in DR patients than in healthy controls (Zhou et al., 2021). Carnosine, succinate, and nicotinic acid exhibit strong antioxidant capacity by scavenging reactive oxygen species (ROS) (Ma et al., 2020b; Murthy et al., 2021; Wang et al., 2021a); lower levels of these can result in excessive ROS triggering a series of pro-inflammation cascades promoting DR. Carnosine supplementation can alleviate diabetic complications and reduce some age-related neurological diseases (Cararo et al., 2015; Tonade and Kern, 2020). Furthermore, D-proline, a nutrient for retinal pigment epithelium, was found lower in DR patients than in DM patients (Tonade and Kern, 2020). Aberrant metabolites in the gut can further remodel microbiota toward maleficent bacteria. In DM, this may increase detrimental or decrease beneficial metabolites in the gut, which can transport to the retina through microvessels initiating DR.

The BAs metabolism was found disturbed in DR mice. It was shown that intermittent fasting may prevent retinopathy, which was accompanied by a significant increase in taurochenodeoxycholate and tauroursodeoxycholate (White et al., 2018). Ursodeoxycholic acid and tauroursodeoxycholate are important secondary BAs that exert multiple hormonal regulatory roles by targeting TGR5 and are structurally modified by microbiota. Recent studies showed that ursodeoxycholic acid and tauroursodeoxycholate may benefit DR by reducing oxidative stress and apoptosis. Tauroursodeoxycholate treatment slowed early changes in visual function in STZ-induced diabetic mice (Bikbova et al., 2015; Chung et al., 2017).

### Bacterial metabolites affect diabetic cardiovascular diseases

In addition to the gut microbiota composition, microbiota metabolites, genes, transcripts, and proteins also contribute to CVD development. IS and PS were found to be associated with aortic calcification and arterial stiffness, which contributed to the progression of CVD (Barreto et al., 2009). Although elevated levels of IS and PS were observed in T2DM patients, the other changes in T2DM with CVDs are unknown. SCFAs play vital roles in energy metabolism and homeostasis that majorly contributes to CVD. A fiber-rich diet with high levels of SCFAs can promote intestinal gluconeogenesis and accelerate insulin secretion by regulating glucagon-like peptide 1 (Tolhurst et al., 2012; den Besten et al., 2015; Hernandez et al., 2019). The abundance of SCFAs showed a negative correlation with CVD mortality and major adverse cardiovascular events (Vijay et al., 2021). Acetate supplementation reduced cardiac fibrosis and left ventricular hypertrophy in hypertensive mice (Marques et al., 2017).

The most coveted microbial metabolite is TMAO, which is a phosphatidylcholine-derived metabolite and oxidized by microbial metabolite trimethylamine (TMA) through flavin-containing monooxygenase 3 in the liver. TMAO exhibited a strong association with increased risk of CVD and therefore has been recognized as a risk biomarker of CVD (Tang et al., 2013; Francois et al., 2018). A study showed that dietary nutrients that can generate TMAO promoted macrophage foam cell formation and increased the risk for aortic atherosclerotic plaque formation (Koeth et al., 2013). In DM, higher levels of plasma TMAO showed a positive association with mortality, CVD, and renal dysfunction (Zhu et al., 2016; Winther et al., 2019). CVD with DM is more likely to have higher TMAO levels (Senthong et al., 2021). Li et al. (2022) showed that plasma TMAO levels were correlated with platelet aggregation and the mean amplitude of glycemic excursions in DM patients. Diabetic rats fed with choline showed increased levels of TMAO and platelet aggregation, which were abolished upon receiving oral antibiotics. Similarly, Tang et al. (2017) showed higher levels (up to threefold) of TMAO in diabetic patients with an increase in 3-year major adverse cardiac events, and a 3.6-fold increase in 5-year mortality risks independent of traditional risk factors, renal function, and glycemic control. Inversely, FMO3 knockdown in insulin-resistant mice prevented the development of atherosclerosis, indicating that targeting FMO3 or TMAO may prevent diabetes-associated CVD (Miao et al., 2015). TMAO may increase CVD by promoting cholesterol accumulation through the upregulation of scavenger receptor A1 inducing endoplasmic reticulum stress and overproduction of ROS. This enhances platelet hyper-reactivity by arousing intracellular Ca^2+^ promoting the development of CVD (Zhu et al., 2016; Chen et al., 2017; Shih et al., 2019).

## Compromised intestinal barrier and aberrant inflammation in diabetic complications

Altered gut microbiota-mediated metabolic dysfunction majorly involves the perturbation of the intestinal barrier and immune system. The gut barrier, including the luminal mucus, gut epithelial, and internal layers, acts as physical and immunological protection against luminal microorganisms or damaging agents from the systemic circulation, which limits host contact with noxious luminal antigens. Cells of the adaptive immune system and pattern recognition receptors including Toll-like receptors (TLRs) and nucleotide-binding oligomerization domain-containing proteins (NODs) play a vital role in maintaining intestinal barrier and remodeling gut microbiota (Lavelle et al., 2009). Upon stimulation from pathogen-associated microbiota or their metabolites, Th17 cells or type3 innate lymphoid cells in the intestinal laminapropria secret IL-17 and IL-22. The elevated levels of IL-17 and IL-22 facilitate intestinal epithelial cells to produce cytokines and chemokines that induce T-cell immune responses or deliver antigens to antigen-presenting cells. This further contributes to antigen-specific IgA responses and the oral tolerance to food antigens. Conversely, activated T cells promote antimicrobial molecules to regulate pathogenic opportunistic microbes (Atarashi et al., 2015; Sano et al., 2016). The abnormal activation of TLRs or the NODs signaling pathway activated by the gut microbiota can overproduce proinflammatory cytokines, such as tumor necrosis factor α (TNFα), Interleukin-1β (IL-1β), and interferon (IFN)-γ, which are the crucial factors in diminishing epithelial barrier function (Capaldo et al., 2012).

**FIGURE 2:**
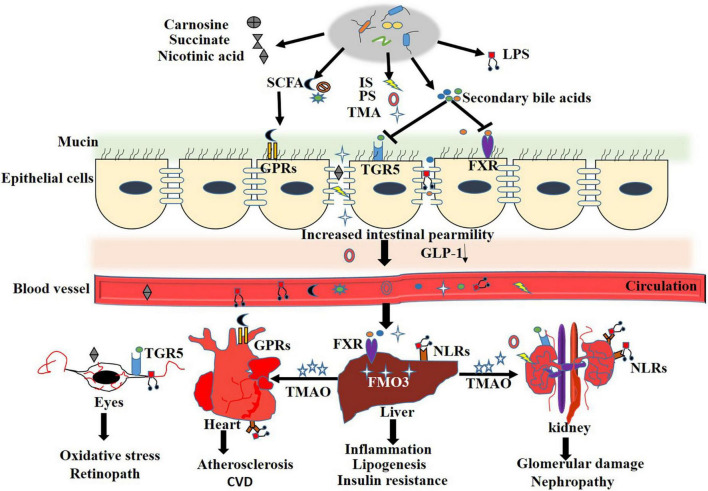
Mechanisms of gut microbiota in regulation of intestinal barrier integrity. During T2DM and the relate complication, disturbed gut microbiota result in overgrowth of pathogenic bacterium, increased LPS and aberrant metabolites. The microbe-associated molecular patterns (MAMPs) and detrimental metabolites activate TLRs or NODs signaling pathway, change intestinal immune landscape toward a pro-inflammatory phenotype by overproducing of pro-inflammatory cytokines, such as tumor necrosis factor α (TNFα), Interleukin-1β (IL-1β) and interferon (IFN)-γ, coupled with a loss in barrier protective cytokines interleukin (IL)-17, IL-22 and epithelial mucin. All these contribute to the degradation of epithelial tight junction proteins and increased intestinal permeability. Compromised intestinal barrier leads to abnormal influx of immune-stimulatory microbial products and systemic spread of enteric pathogens and LPS in metabolic tissues.

Normal gut barrier function is important for maintaining the symbiotic relationship between microbes and host immune cells. Defective gut epithelial barrier function in combination with immune dysregulation contributes to the pathophysiology of various gastrointestinal and extra-intestinal diseases including celiac disease, diabetes, non-alcoholic fatty liver disease, and insulin resistance (Gueddouri et al., 2022). Extensive reviews about the relationship between the gut barrier and metabolic diseases have been published elsewhere (Winer et al., 2016; Akdis, 2021). In this section, we will review the alterations to the gut barrier and the mechanism that contribute to diabetic complications (Figure 2).

### Compromised intestinal barrier and aberrant inflammation in diabetic nephropathy

T2DM is characterized by a compromised intestinal barrier and elevated LPS levels, which contribute to systemic and local low-grade inflammation, thereby promoting the progress of diabetes and subsequent DN (Zihan and Feng, 2016; Feng et al., 2019). LPS administration was shown to enhance urinary albumin excretion and renal injury (Sawa et al., 2014; Lindfors et al., 2021). Several studies showed the increased intestinal permeability and elevated systemic LPS levels in the DN mice model; notably, improving the gut barrier alleviated DN (Feng et al., 2019; Li et al., 2020a; Gao et al., 2021). A study showed that Akkermansia muciniphila, a mucin-degrading bacterium important for maintaining gut integrity, was reduced in DN, while some other intestinal barrier destructive bacteria were increased (Li et al., 2020b). Gut microbial metabolites SCFAs play vital roles in regulating the gut barrier by interacting with intestinal epithelial and immune cells (Ghosh et al., 2021). Recent research showed that butyrate improved the gut barrier function by increasing the expression of mucin2 and claudin and inhibiting the activation of NLRP3 inflammasome and autophagy (Feng et al., 2018; Diao et al., 2019). Similarly, in DN rats, Gonzalez et al. (2019) showed butyrate ameliorated insulin resistance and renal failure by improving intestinal permeability and upregulating mucin expression. Tang et al. (2022) also showed that butyrate reversed renal function by improving the intestinal barrier function via the FFA2 receptor-mediated PI3K/Akt/mTOR pathway in db/db mice. Snelson et al. (2021) revealed that a compromised intestinal barrier increases the risk of kidney disease progression in a diabetic leptin receptor-deficient (Leprdb/db) mice. They first showed that feeding Sprague-Dawley rats with a thermally processed diet (the food processed under heat conditions that generate advanced glycation end-products) increased chemokine monocyte chemoattractant protein-1 levels and incited innate immune complement activation. The process was accompanied by increased intestinal permeability and redistribution of the gut microbiota. Next, they found that supplementing Leprdb/db mouse with high resistant starch fiber diet targeting the gut microbiome maintained gut barrier integrity and alleviated kidney disease via the suppression of complement C5a (Snelson et al., 2021). Once the intestinal barrier is damaged, the violated microorganisms and metabolites elicit the innate immune response by triggering the pattern recognition receptors, producing interleukin-6(IL6), TNFα, and TGFβ in glomeruli, and promoting renal abnormalities (Anders and Lech, 2013). Activation of TLRs and NOD-like receptors (NLRs) in kidney tissues is the pivotal mechanism of gut microbiota exerting an effect on the progression of DN, especially TLR2, TLR4, and the NLRP3 inflammasome. Blocking TLR2/TLR4 signaling pathways or suppressing renal NLRP3 inflammasome activation in diabetic mice decreased urinary albumin excretion and ameliorated diabetes-associated nephrotoxicity (Sridevi et al., 2011; Kim et al., 2013; Miao et al., 2013). Thaiss et al. (2018) showed that hyperglycemia-mediated gut barrier disruption increased the risk of mucosal infection and systemic inflammation. The gut barrier disruption can lead to an abnormal influx of immune-stimulatory microbial products and systemic spread of enteric pathogens in metabolic tissues, triggering DN.

### Compromised intestinal barrier and aberrant inflammation in diabetic retinopathy

Although diabetes is characterized by a compromised intestinal barrier, such changes in DR have not been examined in detail. The first direct evidence for a compromised intestinal barrier in DR was shown by Beli et al. (2018) showed that bacterial products peptidoglycans were increased in DR mice indicating increased gut permeability. However, intermittent fasting prevented retinopathy with a decrease in gut permeability. Preclinical DR begins with the initial diagnosis of diabetes. Therefore, a compromised intestinal barrier and high levels of LPS may be important players in the progression of DR. A cross-sectional study showed that serum intestinal fatty acid-binding protein levels were higher in patients with retinopathy than those without it (Wang et al., 2021b). Individuals with severe DR exhibited high levels of LPS activity (Simonsen et al., 2020). DR is a progression of immune dysregulation. The excess systemic LPS and detrimental microbial metabolites overwhelm the counterbalance immune regulating systems, promoting microglia activation and immune response through the cross-activation of immune-competent microglia (Steinle, 2020). Macroglia in the retina shift from anti-inflammatory response to pro-inflammatory response in DR. Astrocytes and Müller cells that participate in maintaining the blood-retinal barrier (BRB) also showed alterations during the development of DR (Schellini et al., 1995; Fehér et al., 2018). The retinal pigment epithelium forms the inner and most-proximal barrier of BRB. During the progression of DR, retinal pigment epithelium is dysfunctioned due to the disturbed environment, such as a decrease in antioxidant metabolite carnosine and D-proline. IL-1β, TNFα, and monocyte chemoattractant protein-1 also show an increase during the DR progression. The fire inflammation induced by the perturbative environment disrupts BRB recruiting perivascular macrophages (Capozzi et al., 2016). However, direct evidence highlighting the mechanisms of the compromised intestinal barrier in DR is yet absent.

### Compromised intestinal barrier and aberrant inflammation in diabetic cardiovascular diseases

Despite compromised intestinal barrier being the feature of T2DM, only a few studies suggest its direct role in diabetes-associated CVDs. The presence of blood microbiota in CVD patients indicated compromised gut permeability (Velmurugan et al., 2020). Damaged intestinal barrier and increased circulating LPS were found in atherosclerotic and heart failure patients (Peschel et al., 2003; Fatkhullina et al., 2018). Intestinal barrier dysfunction was found associated with the pathogenesis of subclinical vascular disease and CVD in children with parentally acquired HIV (Sahera et al., 2019). IL-22 has been shown to play a vital role in maintaining the intestinal barrier and production of antimicrobial peptides. Intestinal expression of IL-22 was reduced in atherosclerotic patients. Microbiota depletion or IL-22 supplementation reduced inflammation and ameliorated atherosclerosis by improving the gut barrier and restraining the expansion of pro-atherogenic microbiota (Fatkhullina et al., 2018). An impaired intestinal barrier allows microbiota and microbial-associated molecular patterns including LPS or peptidoglycan to translocate into the systemic circulation, which are then recognized by receptors like TLRs and NLRs in the blood vessel endothelium and cardiomyocytes. LPS induces endothelial cell damage, stimulating the release of superoxide anions, IL-1β, and TNFα, subsequently promoting the formation of foam cells by activating TLR4 (Wiedermann et al., 1999; Howell et al., 2011; Kiechl et al., 2013). Antibiotic treatment and depletion of TLR4 can improve vascular dysfunction and alleviate aortic plaque (Michelsen et al., 2004). Nonetheless, so far, little is known about the intestine and system immune response induced by gut microbiota in diabetes associated CVDs.

## Microbiota targeted therapies

### Diet therapies

Diet changes are popular to manage obesity-related insulin resistance. A change from a low-fat and high-fiber diet to a high-fat and high-sugar diet causes dramatic dysbacteriosis, especially causing the irreversible loss of important taxa such as Bacteroidota, which accelerates disease progression by destroying gut barrier and increased susceptibility to infection (Sonnenburg et al., 2016; Kreuzer and Hardt, 2020). A western-style diet shapes the evolution of Bacteroid esthetatiotaomicron favoring the consumption of host mucus (Dapa et al., 2022). Conversely, a high-polysaccharide or fibers rich diet increases the SCFA-producing strains promoting SCFAs concentration, which can alleviate metabolic disease-associated outcomes (Liping et al., 2018; Kjølbæk et al., 2019). Administration of dietary fibers in T2DM patients showed improvement in blood-glucose regulation by activating glucagon-like peptide-1 secretion, impelling fibers as a novel ecological approach for managing T2DM (Liping et al., 2018). Compelling evidence from various epidemiological, preclinical and clinical trials has revealed that dietary fibers can be potential formulations for the treatment of CVD, DM, and obesity by modulating microbiota and metabolites. Furthermore, innovative dietary strategies such as intermittent fasting and protein restriction have recently emerged as popular approaches (Mattson et al., 2016). Twenty eight-days of intermittent fasting in diabetic mice alleviated behavioral impairment via the microbiota-metabolites-brain axis (Liu et al., 2020). In T2DM patients, intermittent fasting was shown to increase insulin resistance (Jørgensen et al., 2007); however, such studies are limited. Horne et al. (2020) suggested that T2DM patients should not adopt intermittent fasting without the involvement of their physicians.

### Probiotics and engineered microbes therapies

A potential strategy to combat metabolic syndrome is the use of probiotic microbes such as Bifidobacterium, Lactobacillus, and *Saccharomyces* spp., which have also been shown to improve insulin resistance and glucose metabolism in T2DM patients (Simon et al., 2015). However, some studies in humans and animals showed that probiotic therapies had no consistent effect in treating obesity-related insulin resistance due to the presence of different bacterial strains impacting therapeutic effectiveness (Rondanelli et al., 2017; Callaway et al., 2019). Interestingly, Akkermansia muciniphila so far not a marketed probiotic strain, exhibited enormous potential in improving metabolic parameters in obese and diabetic individuals. Administration of Akkermansia muciniphila reduced diabetes incidence by remodeling gut microbiota and controlling islet autoimmunity in NOD mice. It is suggested that Akkermansia muciniphila improves the intestinal barrier and reduces colonic inflammation by elevating the expression of Reg3γ. Since inflammatory stress in the colon promotes islet autoimmunity activity, lowering inflammatory stress in the colon lowers the levels of systemic endotoxins, TLR2, and TLR4 in islets. This promotes Foxp3 + regulatory T cells in islets and IL-10 and transforming growth factor-beta in pancreatic lymph nodes, controlling islet autoimmunity (Hänninen et al., 2018). Further research is needed to select safer microbes that can easily colonize the host and favorably affect gut microbiota and metabolic homeostasis. Some exogenous probiotics can be inhabited in the intestine, enhancing microbiota antibiotic resistance and translocation into the circulatory system (Rozman et al., 2019; Ma et al., 2020a). Some prebiotics, such as non-digestible polysaccharides, inulin, and galactose, were found beneficial in metabolic diseases, which improved the gut barrier and reduced inflammatory markers (Everard et al., 2011; Dewulf et al., 2013). Furthermore, symbiotic therapy (a combination of various probiotic strains and/or prebiotics) can favor the establishment of beneficial microbial species and has been found successfully lower the levels of blood glucose and systemic inflammation (Panigrahi et al., 2018; Ke et al., 2019). Genetically engineered microbes are another interesting targeted therapeutic avenue. Several N-acyl-phosphatidy lethanolamines and glucagon-like peptide-1 secreting engineered microbes including Lactobacillus gasseri and *E. coli.* Nissle 1917 have been used for T1DM and T2DM therapy, which can offer an enormous advantage in improving insulin resistance and glucose homeostasis (Chen et al., 2014; Duan et al., 2015). However, whether the genetically modified microbes can be directly diverted to the human gut is an unsettled scientific issue.

### Fecal microbiota transplantation

Fecal microbiota transplantation has been found an effective treatment against chronic infections caused by Clostridioidesdifficile. However, FMT in metabolic dysfunctions yielded inconsistent results. FMT from lean donors temporarily improves insulin resistance in obese metabolic syndrome individuals (Kootte et al., 2017). A recent study showed that combining FMT with lifestyle intervention can improve lipid profile and liver stiffness in T2DM obese patients (Ng et al., 2021). Conversely, another study showed that administration of FMT capsules in obese adults did not clinically change the glucose metabolism (Yu et al., 2020). These discrepancies may be due to a large heterogeneity in FMT donor and processing of FMT. Although FMT is a safe and well-tolerated procedure, a recent report showed that two recipients died from *E. coli* infection transferred during FMT (Defilipp et al., 2019). Thus, serious attention must be paid to donor-acceptor compatibility, the suboptimal match between FMT donor and recipient, preselecting FMT donors, dietary control for stool graft colonization and survival, and optimal anaerobic handling of donor feces (Hanssen et al., 2021).

## Conclusion

Overwhelming advances within the microbiome field have made it evident that microbiota plays a vital role in the development of T2DM and the associated complications by producing metabolites, changing the host gut barrier, and/or directly triggering host reactions. However, only a few findings could be validated or translated to clinical practice. Since the development of T2DM and related complications is a turbulent process, it may also result in oscillatory microbiota. There is a great lack of knowledge about the change of microbiota at the preliminary stage that leads to T2DM complications. Future work is needed on the both short and long-term dynamics of the intestinal microbiome and not just the snapshots of microbiome landscapes. Furthermore, microbiome structure and functionality can vary at evolution levels, which can also contribute to heterogeneity in different reports. Therefore, massive and complex inter-individual variations in the gut microbiome should be seriously taken into account. Unbiased large-scale omics should be conducted to identify critical microbes and metabolites that are concordant in different studies and can ameliorate metabolic syndrome.

The examination of gut microbiota in T2DM-related complications mostly focuses on the microbiome features involving targeted or untargeted metabolite profiling. However, these alone cannot explain the changes during the disease, which can also be directly regulated at the microbiome transcriptional and translational levels. So far, such knowledge is scarce. Thus, uncovering the total interactions between the microbiome and disease is still largely unclear and needs significant collective efforts. So far, compromised intestinal barrier and elevated circulating LPS seems to be the common feature of T2DM-related complications highlighting the importance of crosstalk between intestinal and systemic immune systems. Future work must be done to explore the intestinal immune landscape to better understand activated pathways, associated antigen receptors, and the immune cell-microbiota axis in T2DM-related complications.

## Author contributions

RY conceived the study and revised the manuscript. QL, ZL, and XY wrote the manuscript. AS and YH draw mechanism diagram and performed the constructive discussions. All authors contributed to the article and approved the submitted version.
